# Diagnostic Accuracy of Sonazoid-Enhanced Ultrasonography for Detection of Liver Metastasis

**DOI:** 10.3390/medsci13020042

**Published:** 2025-04-09

**Authors:** Anas Elgenidy, Khaled Saad, Reda Ibrahim, Aya Sherif, Taher Elmozugi, Moaz Y. Darwish, Mahmoud Abbas, Yousif A. Othman, Abdelrahman Elshimy, Alyaa M. Sheir, Dina H. Khattab, Abdallah A. Helal, Mario M. Tawadros, Osama Abuel-naga, Hazem I. Abdel-Rahman, Doaa Ali Gamal, Amira Elhoufey, Hamad Ghaleb Dailah, Rami A. Metwally, Noran ElBazzar, Hashem Abu Serhan

**Affiliations:** 1 Faculty of Medicine, Cairo University, Cairo 12613, Egypt; 2 Pediatric Department, Faculty of Medicine, Assiut University, Assiut 71516, Egypt; 3 Faculty of Medicine, Beni Suef University, Beni Suef 62511, Egypt; 4 Faculty of Medicine, Benghazi University, Benghazi 18251, Libya; 5 Faculty of Medicine, Fayoum University, Fayoum 63514, Egypt; 6 Department of Radiology, Detroit Medical Center, Wayne State University, Detroit, MI 48201, USA; 7 Faculty of Medicine, Alexandria University, Alexandria 21526, Egypt; 8 Faculty of Medicine, Ain Shams University, Cairo 11591, Egypt; 9 Department of Radiology, Faculty of Medicine, Ain Shams University, Cairo 11591, Egypt; 10 Clinical Oncology Department, Faculty of Medicine, Assiut University, Assiut 71516, Egypt; 11 Department of Community Health Nursing, Alddrab University College, Jazan University, Jazan 45142, Saudi Arabia; 12 Department of Community Health Nursing, Faculty of Nursing, Assiut University, Assiut 71111, Egypt; 13 Research and Scientific Studies Unit, College of Nursing, Jazan University, Jazan 45142, Saudi Arabia; 14 Department of Internal Medicine, Benha University, Benha 13511, Egypt; 15 Department of Ophthalmology, Hamad Medical Corporation, Doha 3050, Qatar

**Keywords:** hepatocellular carcinoma, liver metastasis, Sonazoid, ultrasonography

## Abstract

Purpose: To evaluate the potential clinical role and reliability of Sonazoid-enhanced ultrasound (SEUS) as a diagnostic tool for liver metastatic lesions. Methods: An extensive literature search was conducted across five electronic databases, PubMed, Scopus, Embase, Cochrane Library, and Web of Science, from their inception up to January 2024 to identify all studies evaluating the use of Sonazoid-enhanced ultrasonography for detecting hepatic metastases. A meta-analysis was performed to assess diagnostic accuracy using the Meta-DiSc 2.0 software. Results: A total of 31 studies were included, 16 of which were eligible for meta-analysis and diagnostic test accuracy evaluation. A total of 13 studies in the meta-analysis evaluated the diagnostic accuracy of contrast-enhanced ultrasound (CEUS) for 1347 metastatic and 1565 non-metastatic liver lesions. The pooled sensitivity and specificity for CEUS were 0.88 (95% CI: 0.82–0.92) and 0.92 (95% CI: 0.84–0.96), respectively. The combined positive likelihood ratio, negative likelihood ratio, and diagnostic odds ratio were 11.89 (95% CI: 5.42–26.09), 0.12 (95% CI:0.08–0.19), and 91.99 (95% CI: 32.15–263.17), respectively. Additionally, four studies of the meta-analysis assessed the diagnostic performance of contrast-enhanced intraoperative sonography (CE-IOUS) in detecting 664 metastatic and 246 non-metastatic liver lesions. The pooled sensitivity and specificity for CE-IOUS were 0.93 (95% CI: 0.82–0.97) and 0.84 (95% CI: 0.65–0.93), respectively. The aggregated positive likelihood ratio, negative likelihood ratio, and diagnostic odds ratio were calculated as 5.95 (95% CI: 2.32–15.25), 0.07 (95% CI: 0.02–0.24), and 77.68 (95% CI: 10.33–583.86), respectively. Conclusions: CE-IOUS and CEUS are reliable approaches for diagnosing liver metastatic lesions. CE-IOUS, in particular, exhibits higher accuracy in identifying liver metastatic lesions, indicating its potential effectiveness in clinical practice.

## 1. Introduction

The liver is one of the most common sites for tumor cells due to its distinctive and diverse architectural composition [1,2] It is characterized by a unique microvascular anatomy and dual blood supply system through the portal vein and the hepatic artery, which renders the liver a prevalent site for organ-specific metastasis [3,4]. Among the metastatic cancer patients reported in the SEER database between 2010 and 2015, the one-year survival rate was 15.1% for patients with liver metastases, compared to 24.0% for patients without liver metastases. Breast cancer and colon cancer are the most common primary diagnosis with liver metastasis in female and male patients, respectively, within the age range of 20–50 years [5]. At least 25% of patients with colorectal cancer (CRC) develop liver metastases during the disease [6,7]. Nearly 75% of patients with colorectal liver metastases who undergo hepatic resections will develop recurrence, and 65 to 85% of these recurrences occur in 2 years [8,9]. This happens mainly due to occult liver metastasis that cannot be detected at the time of hepatic resection. Accurate diagnosis of liver metastasis has an influential role in designing the management plan as it provides information about the site, size, and vascular relation of the hepatic lesion. Different imaging modalities can be used for this purpose, including CT, MRI, PET, and grayscale ultrasonography (US) [10].

Sonazoid, a second-generation contrast agent, received clinical approval in Japan in 2007. It has gained widespread adoption in Japan and South Korea due to early regulatory approvals and its inclusion in national clinical guidelines, facilitating its integration into routine diagnostic practices [11]. The agent consists of a perfluorobutane microbubble surrounded by a phospholipid monolayer envelope. It has a unique ability to accumulate in the Kupfer cells with a 99% phagocytic ratio [12]. This leads to prolonged hepatic parenchyma enhancement during CEUS examinations and washout of hepatic malignancies during the Kupffer phase, highlighting the defect by enhancing the surrounding tissue [13,14,15]. Additionally, Sonazoid allows real-time visualization of arterial phase hyperenhancement (APHE), a feature not achievable by other imaging techniques [11].

Contrast-enhanced ultrasound (CEUS) combines conventional ultrasound (US) with microbubble contrast agents to visualize blood flow and tissue perfusion, enabling real-time dynamic imaging without ionizing radiation, which is particularly useful for detecting liver metastases. Contrast-enhanced intraoperative ultrasound (CE-IOUS) is used during surgery to enhance the visualization of tissue vascularity and tumor margins in real-time. Intraoperative ultrasound (IOUS) offers more accurate tumor size assessment and its relationship with surrounding tissue, as preoperative US is limited by percutaneous and angled application. IOUS is performed directly on the organ surface, and CE-IOUS improves tumor identification [16,17].

Multiple studies have investigated Sonazoid-enhanced ultrasound (SEUS) and its ability to detect hepatic metastases by producing functional images of the Kupffer cells, with varying diagnostic performances reported [18,19]. Our meta-analysis highlights Sonazoid’s advantages over traditional contrast agents, providing a longer enhancement in the liver due to its Kupffer cell uptake. This extended contrast duration enables a more comprehensive evaluation of liver parenchyma. SEUS is non-invasive, radiation-free, and allows repeat examinations without significant risk [19,20,21,22,23]. These features make it a valuable tool for diagnosing and monitoring liver tumors, particularly in patients unsuitable for other imaging modalities. In this meta-analysis, we aim to assess the clinical role and reliability of SEUS in diagnosing liver metastatic lesions.

## 2. Methods

The study was conducted in accordance with the PRISMA guidelines [24]. Our study was registered in PROSPERO, “International Prospective Register of Systematic Reviews”, under number: CRD420250656049 https://www.crd.york.ac.uk/PROSPERO/view/CRD420250656049 (accessed on 24 February 2025).

### 2.1. Search Strategy and Study Selection

A comprehensive literature search was performed across five electronic databases—MEDLINE via PubMed, Scopus, Embase, the Cochrane Library, and Web of Science—from their inception until January 2024 to identify pertinent studies. The complete search strategy employed is detailed in Supplementary Table S1. After removing duplicate records, two authors independently screened the titles and abstracts of the retrieved studies based on predefined eligibility criteria. The list of potentially eligible studies was then subjected to further evaluation by the same two authors. Studies that met the inclusion criteria were selected, and any disagreements were resolved through full-text assessment. Additionally, a manual search was conducted independently by two authors by reviewing the reference lists of the included articles and relevant literature reviews to identify additional pertinent studies.

### 2.2. Eligibility Criteria

Studies that met the following criteria were included: (a) studies that evaluated liver metastasis imaging and diagnosis using contrast-enhanced ultrasonography with Sonazoid. We included studies with the following designs: prospective and retrospective cohort, case–control, and cross-sectional studies. (b) Study publication should be in peer-reviewed international journals indexed in Scopus, WOS, PubMed, Embase, or Cochrane, while (c) no limitations placed on language or study type. However, we excluded the following: (a) duplicate publications, (b) review articles, meta-analyses, case reports, conference abstracts, replies, letters to editors, book chapters, and comments, as well as (c) animal and in vitro studies. Studies not related to the outcome of interest in this investigation are shown in Supplementary Table S2.

### 2.3. Data Extraction

A team of nine authors was responsible for extracting data about baseline characteristics and outcomes included in the analysis. To resolve any conflicts, the data were reassessed by a different author who was not involved in the initial extraction process. The baseline characteristics data, such as author, publication year, study design, country, participants’ characteristics (age and sex), type and number of lesions, aim, results, and conclusion, were collected into a pre-piloted Excel spreadsheet. The data extracted for the analysis included the number of cases (true positives [TP] + false negatives [FN]), controls (true negatives [TN] and false positives [FP]), detection rate, positive and negative predictive value, and reference tests used to detect metastatic lesions, such as biopsy, surgery, histopathology, or CT and MRI imaging. The studies that did not provide raw data and only provided sensitivity and specificity were extracted to ensure data consistency and accuracy; two authors carefully studied the completed extraction sheet, resolving any inconsistencies, and authenticated the accuracy of the data.

### 2.4. Critical Appraisal Tool and Risk of Bias Assessment

In this meta-analysis, a comprehensive quality assessment was undertaken to detect any low-quality studies with outlier results that could influence heterogeneity or the overall effect size. The diagnostic accuracy studies included were evaluated using the Quality Assessment of Diagnostic Accuracy Studies 2 (QUADAS-2) tool [25]. This instrument addresses four domains of potential bias: patient selection, index test, reference standard, and flow and timing. The framework was scored via classifying the questions into yes, no, or unclear categories. Unclear means there is insufficient information available to make the judgment.

These questions classified the study into different categories (high, low, and unclear). Regarding the patient selection domain, studies without any reference to the sampling procedure were classified as high risk. Studies that did not report blinding for interpreting reference standard and index test results were classified as unclear. Any conflicts of interest were delegated to the senior author.

For the systematic review studies, we utilized the National Institutes of Health (NIH) quality assessment tool tailored for observational cohort and cross-sectional studies, which encompasses 14 “yes or no” criteria evaluating factors, such as the specificity of the research question, the selection, and characterization of the study population, and the identification and control of confounding variables, among other elements. Six of these criteria—specifically numbers 3, 6, 7, 8, 10, and 14—relating to the measurement of exposure prior to outcome assessment, varying levels of exposure over time, and the sufficiency of the timeframe to observe an effect, did not apply to the studies in question. Question 3 is for cross-sectional studies and has no value for diagnostic test accuracy studies. Questions 6, 8, and 10 are based on relationships with risk factors, and we do not have risk factors in our studies. Question 7 is related to time frame, and, in our studies, we have neither time frames nor long follow-up periods for outcomes. Finally, for question 14, we have no confounding variables in our studies. Consequently, we adjusted the maximum attainable quality score for each study to 8 points. The quality of studies scoring 6–8 stars was considered good, the quality of studies scoring 4–5 stars was considered fair, and the quality of studies scoring 0–3 stars was considered poor quality [26].

### 2.5. Data Analysis

A meta-analysis was undertaken to evaluate diagnostic accuracy using Meta-DiSc 2.0 software [27]. Counts of TP, TN, FP, and FN were derived from sensitivity and specificity data extracted via the RevMan calculator. Aggregated estimates of sensitivity, specificity, positive likelihood ratio (PLR), negative likelihood ratio (NLR), and diagnostic odds ratio (DOR) were computed. A DOR ranging from 2 to 5 signifies moderate diagnostic accuracy, while a DOR exceeding 10 indicates strong diagnostic accuracy. Forest plots were utilized to depict the pooled diagnostic sensitivity and specificity and to illustrate heterogeneity among the included studies. Additionally, the performance of the diagnostic test was assessed by calculating the point estimate and constructing a bivariate summary receiver operating characteristic (SROC) curve. On this curve, the x-axis denotes specificity, the y-axis represents sensitivity, and the diagonal line corresponds to the equilibrium of sensitivity and specificity of the index test.

In the heterogeneity analysis, logit variances of sensitivity and specificity were examined to identify statistically significant variances, indicating substantial heterogeneity in diagnostic accuracy across studies. A variance greater than 0.5 is generally indicative of considerable heterogeneity. Furthermore, the bivariate I-squared index was calculated; values exceeding 50% are typically interpreted as moderate to high heterogeneity, while values above 75% denote high heterogeneity. The median odds ratios for sensitivity and specificity were also evaluated; a higher median odds ratio signifies a stronger association, whereas a lower median odds ratio suggests a weaker association. Moreover, the analysis incorporated the 95% prediction ellipse area, which delineates a region where future sensitivity and specificity values from similar studies are likely to fall with 95% confidence; a larger area indicates greater variability in sensitivity and specificity [28].

## 3. Results

### 3.1. Study Selection

**Figure 1:**
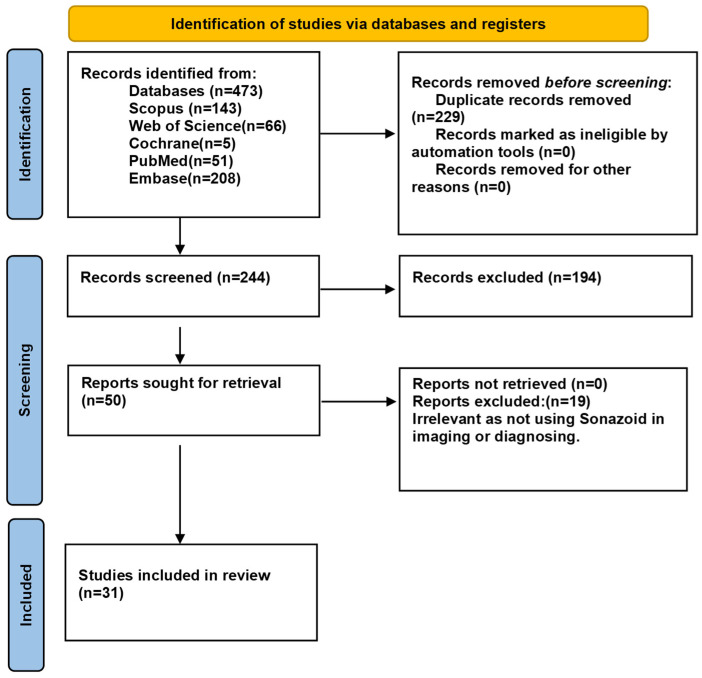
PRISMA flowchart for the search results.

Comprehensive research was completed using the previously mentioned databases and revealed 473 studies. After the removal of duplicates, we were left with 244 studies. The authors conducted title and abstract screening, reducing the number to 50, of which 19 studies were excluded by full-text screening, as shown in Supplementary Table S2. Eventually, 31 studies were included in our systematic review, of which 16 were eligible for meta-analysis [18,19,20,21,22,23,29,30,31,32,33,34,35,36,37,38], because the remaining studies did not provide sufficient data [39,40,41,42,43,44,45,46,47,48,49,50,51,52,53] (Figure 1).

### 3.2. Study Characteristics

The total number of patients in the included studies was 1637, with hepatic metastatic lesions originating from various primary sites. We included 31 studies with different designs, whether prospective or retrospective studies. Most of the included studies were conducted in Japan. Two studies [18,19] were carried out in China, two studies by Edey et al. [40] and Ramnarine et al. [47] were carried out in the United Kingdom, and one study [29] was carried out in Austria. Table 1 displays the characteristics of our included studies.

### 3.3. Meta-Analysis Studies

A meta-analysis of 16 studies [18,19,20,21,22,23,29,30,31,32,33,34,35,36,37,38] was conducted to evaluate using contrast-enhanced imaging modalities for detecting liver metastases. Four studies employed Sonazoid CE-IOUS for colorectal liver metastasis [18,23,37,38], including a prospective study by Li et al. [18] that used CE-IOUS and CEUS modalities to detect metastatic and non-metastatic liver lesions. The remaining 12 studies exclusively utilized CEUS, with 9 focusing on colorectal liver metastases using Sonazoid CEU [19,20,22,29,30,31,34,35]. Among these, Mishima et al. [22] specifically investigated breast cancer liver metastases, while Ishikawa et al. [30] and Minga et al. [34] applied CEUS to detect liver metastases from pancreatic cancer. These studies included prospective and retrospective designs, highlighting the utility of CEUS and CE-IOUS in diagnosing liver metastases across various cancer types.

### 3.4. Quality of Studies

**Figure 2:**
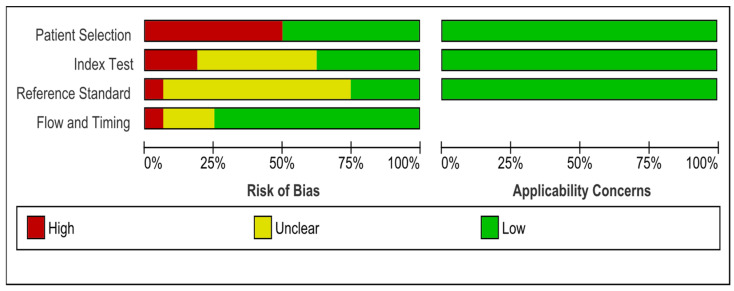
The methodological quality assessment summary of the included studies.

**Figure 3:**
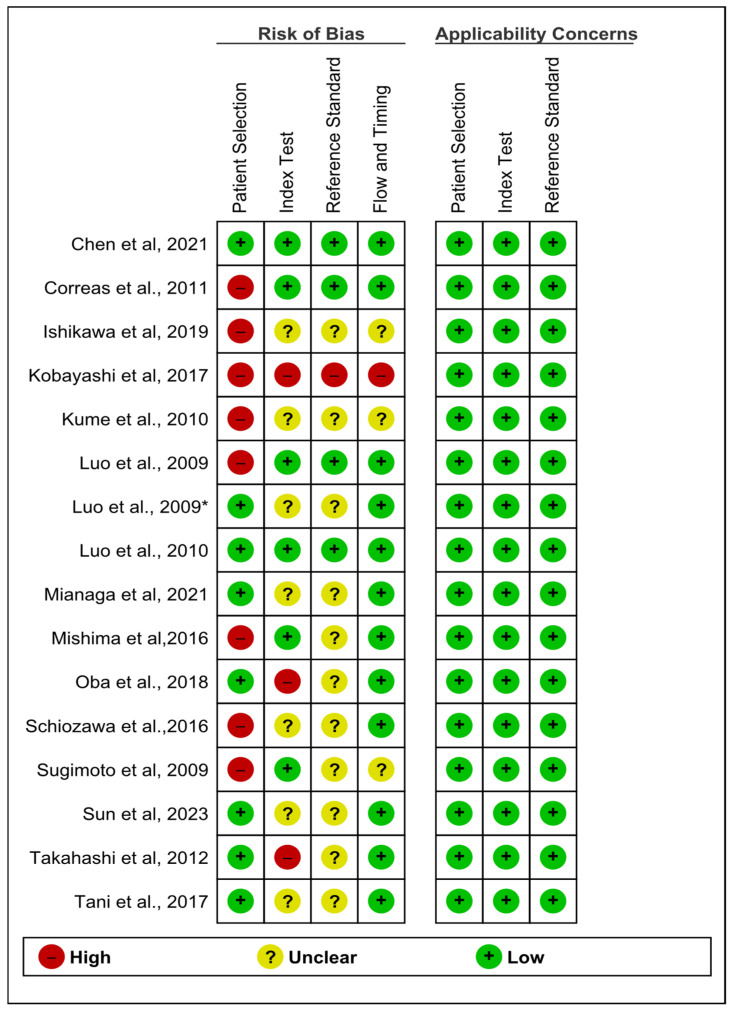
The quality assessment graph of the included studies [18,19,20,21,22,23,29,30,31,32,33,34,35,36,37,38].

Quality assessment was performed using both the NIH scale and the QUASDAS tool. The NIH scale was used for all cohort studies that were not compatible for analysis; overall, 15 of the 31 included studies were assessed using the NIH tool, 11 of which [39,40,41,43,45,46,47,49,50,51,52] were rated as having good quality, while 4 [42,44,48,53] were rated fair, and no study was rated poor (Supplementary Table S3). The QUADAS-2 tool was used for the 16 studies included in the diagnostic accuracy analysis [18,19,20,21,22,23,29,30,31,32,33,34,35,36,37,38]. The assessment of the patients’ selection showed high bias in eight studies [20,22,29,30,31,33,35,36] because the participants were not consecutively selected. For the index test assessment, seven studies were considered low bias [18,21,22,29,33,36], while three studies were considered high bias [20,23,38]. The remaining studies were unclear due to the lack of essential data. Applicability was of low concern for all studies in the patient selection and index test domains. The risk of bias due to the reference standard test was high in only one study [20] (Figure 2 and Figure 3).

### 3.5. Diagnostic Test Accuracy Meta-Analysis for CEUS in Detecting Metastatic Lesions

**Figure 4:**
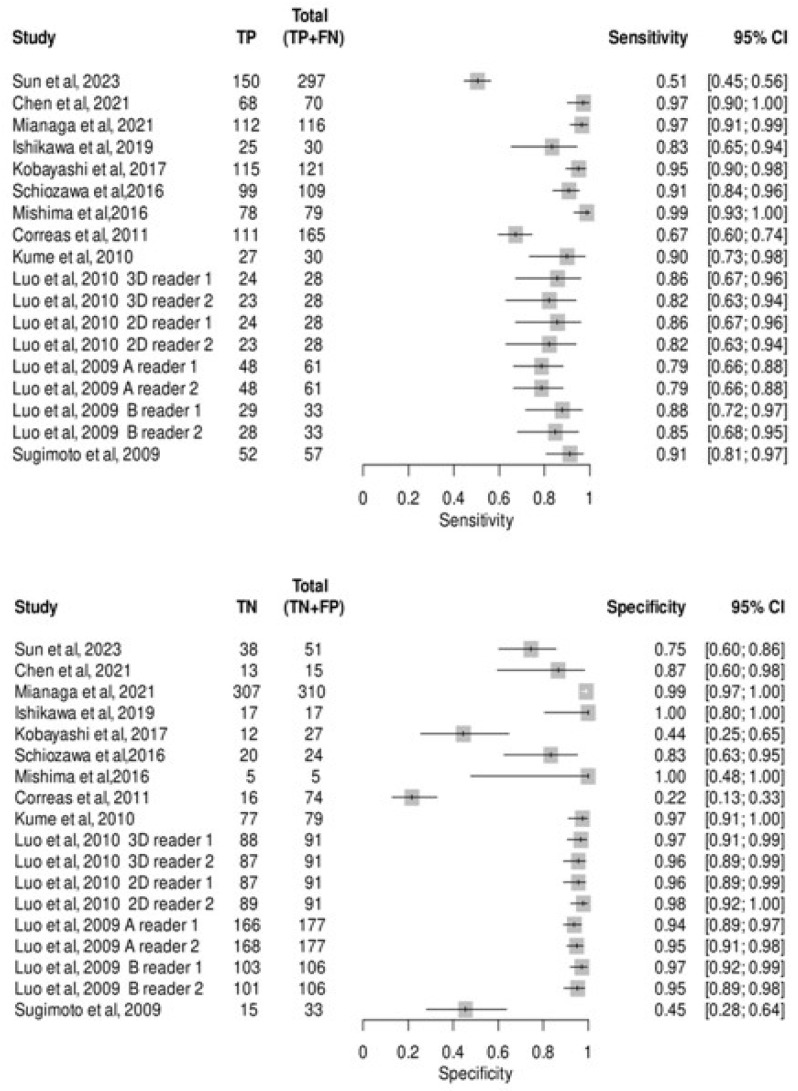
Meta-analysis for CEUS sensitivity and specificity in detecting metastatic lesions [18,19,20,21,22,29,30,31,32,33,34,35,36].

A total of 13 studies [18,19,20,21,22,29,30,31,32,33,34,35,36] assessed the diagnostic accuracy of CEUS in 1347 metastatic liver lesions and 1565 non-metastatic liver lesions. The pooled sensitivity and specificity of CEUS were 0.88 (95% CI: 0.82–0.92) and 0.92 (95% CI: 0.84–0.96), respectively shown in Figure 4, The pooled positive likelihood ratio, negative likelihood ratio, and diagnostic odds ratio were 11.89 (95% CI 5.42–26.09), 0.12 (95% CI 0.08–0.19), and 91.99 (95% CI 32.15–263.17), respectively. We found heterogeneity among the studies with a bivariate I^2^ test at 0.67, indicating substantial heterogeneity. Sensitivity and specificity had a logit variance of 0.79 and 2.76, respectively, with a mean odds ratio of 2.34 and 4.88. The area of the 95% prediction ellipse using the SROC curve was 0.47 (Supplementary Figure S1).

### 3.6. Diagnostic Test Accuracy Meta-Analysis for CEIOS in Detecting Metastatic Lesions

**Figure 5:**
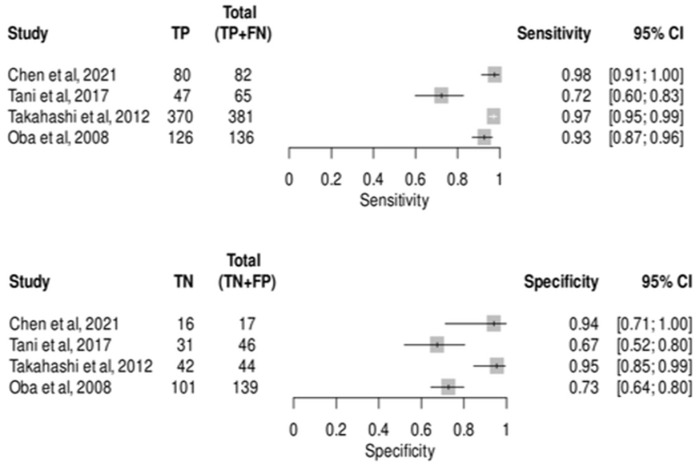
Meta-analysis for CE-IOUS sensitivity and specificity in detecting metastatic lesions [18,23,37,38].

A total of 4 studies [18,23,37,38] assessed the accuracy of CE-IOUS in diagnosing 664 patients with metastatic lesions in the liver and 246 non-metastatic lesions. The pooled sensitivity was 0.93 (95% CI, 0.82–0.97), the pooled specificity was 0.84 (95% CI, 0.65–0.93), as shown in Figure 5, the pooled PLR was 5.95 (95% CI 2.32, 15.25), the pooled NLR was 0.07 (95% CI 0.02, 0.24), the pooled diagnostic odds ratio was 77.68 (95% CI 10.33, 583.86), and the pooled false positive rate was 0.15 (95% CI 0.06, 0.34). The sensitivity of the studies ranged from 72% to 98%, while the specificity ranged from 67% to 95%. We found heterogeneity among the studies with a bivariate I^2^ test at 0.57, indicating moderate heterogeneity. Sensitivity and specificity had a logit variance of 1.13 and 0.8, respectively, with a mean odds ratio of 2.76 and 2.35. The area’s 95% prediction ellipse using the SROC curve was 0.78 (Supplementary Figure S2).

### 3.7. Subgroup Analysis for CEUS in Detecting Metastatic Lesions

We performed a subgroup analysis based on the type of ultrasound to evaluate its accuracy in diagnosing metastatic liver lesions. For the standard CEUS, the pooled sensitivity was 0.89 (95% CI 0.8–0.94), and the specificity was 0.89 (95% CI 0.71–0.96) (Supplementary Figure S3). The pooled positive likelihood ratio, negative likelihood ratio, and diagnostic odds ratios were 8.12 (95% CI 2.84–23.22), 0.12 (95% CI 0.06–0.23), and 67.69 (95% CI 14.82–309.18), respectively.

As for the 3D CEUS modalities, the pooled sensitivity was 0.85 (CI 95% 0.78–0.90), and the specificity was 0.96 (95% CI 0.94–0.97) (Supplementary Figure S4). The pooled positive likelihood ratio, negative likelihood ratio, and diagnostic odds ratio were 22.4 (95% CI, 13.55–37), 0.15 (95% CI 0.1–0.23), and 146 (95% CI 71.14–299.5), respectively. Only one study was conducted for 2D CEUS for metastasis, i.e., by Luo et al. [21], with a sensitivity of 84%, specificity of 97%, and Az value of 0.94 (mean of two readers).

### 3.8. Studies Included in the Systematic Review

Fifteen studies [39,40,41,42,43,44,45,46,47,48,49,50,51,52,53] were included as review studies and not in the meta-analysis (Table 2). Nanashima et al. [45] included patients with both primary and secondary hepatic lesions. Hakamada et al. [42] used Sonazoid CEUS to detect smaller CRLMs that were undetectable with conventional modalities.

## 4. Discussion

In this comprehensive meta-analysis, we evaluated the impact of Sonazoid-enhanced ultrasonography on detecting hepatic metastases, marking the first meta-analysis focused on diagnostic accuracy within this area. Our study revealed that contrast-enhanced intra-operative ultrasonography (CE-IOUS) offers the highest sensitivity (0.93) for detecting liver metastatic lesions. Sonazoid-enhanced ultrasonography (CEUS) also showed encouraging results for detecting HCC and liver metastatic lesions, achieving similar specificity (92%) and nearly equal sensitivity (87% for HCC and 88% for liver metastatic lesions).

Our results suggest that CE-IOUS enhances staging accuracy in patients undergoing resection for CRLM, even after a thorough preoperative assessment. Tumor burden often remains significantly underestimated post-preoperative chemotherapy, requiring adjustments in surgical planning. These insights highlight the critical role of CE-IOUS in identifying “disappearing” metastases. Reports indicate that the prevalence of disappearing liver metastases (DLM) ranges from 9% to 24% [54]. CE-IOUS is more sensitive than palpation and IOUS in detecting DLM, revealing an extra 10–15% of these lesions [37].

Given the accessibility and affordability of ultrasound, our findings suggest that Sonazoid-enhanced ultrasonography could serve as the primary choice for screening the presence of HCC and liver metastatic lesions. The widespread use of CEUS for HCC detection improves the identification of determinants and the prognosis of HCC, thus facilitating targeted interventions for these patients. CEUS offers longer and more stable imaging during the Kupffer phase, which helps detect often overlooked nodules, thereby enhancing CEUS specificity [55]. Furthermore, CEUS is more accurate in detecting HCC during the Kupffer re-injection phase compared to CT/MRI [56]. CEUS had higher specificity and PPV for the diagnosis of liver metastases than EOB-MRI [36]. The increased mechanical index used with CEUS enhances penetration, which helps identify CRLM lesions in the fatty liver [19]. The high specificity of CEUS in detecting liver metastatic lesions provides a reliable alternative to conventional imaging modalities.

Sonazoid is an innovative microbubble contrast agent that generates parenchyma-specific imaging by accumulating within hepatic Kupffer cells, categorizing it as a second-generation agent with sufficient intravascular stability [55,57]. Unique to Sonazoid is its capability for late Kupffer-phase imaging, complementing its early vascular-phase and sinusoidal-phase imaging modalities. In comparison, SonoVue achieves parenchyma-specific contrast by transiently decelerating microbubbles mechanically within the hepatic sinusoids; these agents are minimally phagocytosed by Kupffer cells [12]. While Sonazoid functions similarly to SonoVue, it features an extended half-life exceeding five minutes following intravenous bolus injection. The prolonged duration of approximately 30 min for late Kupffer-phase imaging using Sonazoid appears advantageous for the postoperative detection of suspicious hepatic nodules.

Conventional B-mode US is relatively ineffective at detecting small liver metastases due to their size and the inherent limitations of the imaging technique [36]. In contrast, Sonazoid CEUS significantly enhances detection capabilities, particularly for lesions smaller than 1 cm. Research indicates that Sonazoid CEUS can identify tumors as small as 4 mm, which are often missed by B-mode [31]. Additionally, it has successfully detected occult lesions that were not identified by conventional contrast-enhanced computed tomography (CECT), with many confirmed as true positives [20]. Sonazoid enhances detection capabilities by preferentially accumulating in Kupffer cells, creating a distinct hypoechoic defect pattern for metastases against the hyperechoic liver background during the late Kupffer phase. The prolonged post-vascular phase also allows for a more thorough examination of liver tissue, increasing the chances of detecting subtle lesions [20,32]. CEUS with 3D imaging improves tumor vascularity and morphology characterization, outperforming conventional B-mode ultrasound [21,33]. Additionally, defect reperfusion imaging analyzes the contrast agent’s wash-in and washout patterns to help distinguish benign from malignant lesions [18]. Sonazoid CEUS significantly impacts clinical decision-making by improving surgical planning and enabling better identification of metastases for more complete tumor resections, ultimately enhancing patient outcomes. Its findings can modify treatment strategies, influencing the extent and approach of surgery. Additionally, it helps identify residual tumor cells in liver metastases that may appear absent on CECT, promoting minimally invasive procedures through detailed tumor information [18].

The subgroup analysis examining the accuracy of different CEUS types in diagnosing metastatic liver lesions revealed that 3D CEUS modalities exhibited the highest diagnostic accuracy. This conclusion is based on the higher diagnostic odds ratio observed for 3D CEUS compared to standard and 2D CEUS. The pooled sensitivity and specificity of 3D CEUS modalities were 0.85 and 0.96, respectively. The positive likelihood ratio was 22.4, the negative likelihood ratio was 0.15, and the diagnostic odds ratio was 146. These values indicate that 3D CEUS is highly effective in correctly identifying both the presence and absence of metastatic liver lesions. However, it is important to acknowledge the limitations of this analysis. A key limitation is the small number of studies included for 2D and 3D CEUS, making it difficult to draw definitive conclusions about their performance. Only one study utilized 2D CEUS for metastasis assessment, reporting a sensitivity of 84%, specificity of 97%, and an Az value of 0.94. The limited sample size for these subgroups emphasizes the need for further research to confirm these findings and strengthen the evidence supporting the use of 2D and 3D CEUS in detecting liver metastases.

The methodological strengths in our study can be summarized as follows: A comprehensive search was undertaken across five major databases. Rigorous quality assessment was ensured by using the QUADAS-2 and NIH tools. Clear adherence to PRISMA guidelines was observed. Appropriate statistical analysis was carried out with Meta-DiSc 2.0. Meaningful subgroup analysis was conducted via stratifying by ultrasound techniques. Our meta-analysis includes a larger number of studies that provide deeper insight into the diagnostic accuracy of Sonazoid-enhanced ultrasonography for detecting liver metastatic lesions. However, a clinical study will be required to compare the effects of different contrast agents, such as Sonazoid and SonoVue. Sonazoid’s CE-IOUS intraoperative examination time may be shorter than that of Sonovue.

The substantial heterogeneity reported in the studies is the main intrinsic limitation of this meta-analysis. We addressed the heterogeneity using the methods described by Cochrane, i.e., by conducting sensitivity analysis and subgroup analysis. However, the heterogeneity likely originated from a variety of US equipment, transducer bandwidth, contrast-specific sequences, and true acoustic output power, all of which may have influenced both US and CEUS acquisitions. Some studies failed to identify multiple lesions using CE 3D US and instead evaluated the diagnostic accuracy of CE 3D US for a single lesion, resulting in high heterogeneity among studies [21]. Nevertheless, we addressed the high heterogeneity challenge through subgrouping and sensitivity analyses. We acknowledged it as a limitation in the discussion for future research and considered it while pooling our conclusions. Another limitation is that ultrasonography is a real-time examination with no specific standardization of probe location compared to CT or MRI. The sector sweep of the US probe limits the field of vision, and few anatomical markers are available for reference, making it challenging to appropriately allocate lesions to Chouinard segments. Also, limited geographic diversity (most studies from Japan) a variability in clinical guidelines, and recommendations across different medical societies complicate the assessment of this approach’s economic impact on healthcare systems, indicating that further research is necessary to establish its cost-effectiveness in routine hepatic tumor surveillance, as are standardized protocols to reduce heterogeneity, direct comparisons with other contrast agents (e.g., SonoVue), and larger multicenter prospective studies.

## 5. Conclusions

Sonazoid-enhanced ultrasonography shows promise as an alternative imaging modality for detecting liver metastatic lesions. CE-IOUS demonstrates higher accuracy in detecting liver metastatic lesions, indicating that both CEUS and CE-IOUS hold potential as reliable methods for detecting liver lesions.

## Figures and Tables

**Table 1:** Characteristics of all the included studies in both systematic review and meta-analysis.

Study ID	Country	Design	Age Mean (Range)	Sex (M/F)	Aim	Type of Lesions	Number of Lesions	Reference Test	Conclusion and Results
**Li** **et al., 2021 [18]**	China	Prospective study	59.6 (48.3–70.9)	20/07	To compare the diagnostic performance of CE-IOUS with Kupffer-phase imaging in metastatic liver tumors.	Liver metastasis/others	82/17	88 through histopathology and 11 through various imaging modalities	CE-IOUS with Sonazoid showed high sensitivity (97.5%) and specificity for detecting liver lesions, outperforming MRI (93.3%) and CEUS (97.1%) during the Kupffer phase. It also identified additional lesions, highlighting its potential superiority in diagnosing liver metastases and guiding treatment decisions.
**Luo** **et al., 2009 [32]**	Japan	Retrospective study	62 (47.3–76.7)	90/49	To compare the diagnostic performance of contrast-enhanced three-dimensional ultrasonography to contrast-enhanced three-dimensional computed tomography in detecting liver tumors.	Liver metastasis	33	Biopsy, surgery, or MR imaging	Readers 1 and 2 achieved 83% concordance between US and CT findings in 139 lesions with high inter-modality and inter-reader concordance. Both modalities were equally capable of diagnosis. Contrast-enhanced 3D US holds the potential for characterizing focal liver tumors.
						HCC	77		
**Luo** **et al., 2009 [33]**	Japan	Prospective study	64 (50.6–77.4)	78/64	To evaluate late-phase enhancement patterns of focal liver tumors using Sonazoid-enhanced ultrasonography with intermittent high mechanical index imaging.	Liver metastasis	61	Histopathology	Sonazoid-enhanced ultrasound with high mechanical index intermittent imaging effectively assessed late-phase enhancement patterns of liver tumors. It had good diagnostic accuracy for the distinction between HCC, metastases, hemangiomas, and FNH, with excellent inter-reader agreement, suggesting its utility in the characterization of liver tumors.
						HCC	109	72 lesions by CT and MRI and 37 lesions by histopathology	
**Luo** **et al., 2010 [21]**	Japan	Retrospective and prospective studies	67.4 (61.0–73.8)	Prospective: 90/73, retrospective study: 67/52	To differentiate focal liver lesions based on enhancement patterns using three-dimensional ultrasonography (3D US) with a perflubutane-based contrast agent.	Liver metastasis	28	CT and MRI	Contrast-enhanced 3D ultrasound effectively distinguishes liver tumors with high accuracy, matching or exceeding 2D ultrasound. It provides valuable spatial perspectives, aiding in the differentiation of hepatocellular carcinomas, metastases, hemangiomas, and focal nodular hyperplasia.
						HCC	70		
**Minaga et al., 2021 [34]**	Japan	Retrospective study	69	291/209	To assess Kupffer-phase imaging in CH-EUS for detecting liver metastases from pancreatic cancer, comparing it with B-mode EUS.	Liver metastasis	116	Histopathological findings of EUS-FNA samples and follow-up imaging examinations	CH-EUS with Kupffer-phase imaging had greater diagnostic accuracy (98.4%) for left-lobe liver metastasis in pancreatic cancer patients than CE-CT (90.6%) and FB-EUS (93.4%). It was particularly good at detecting small metastases (<10 mm) and impacted staging in 2.1% of the patients. These findings indicate that CH-EUS is an important tool for pretreatment assessment.
							36		
**Sugimoto et al., 2009 [36]**	Japan	Prospective study	69.5 (59.3–79.7)	21/16	To compare B-mode ultrasonography (US) alone and in combination with B-mode and contrast-enhanced (Sonazoid) late-phase pulse-inversion US for the detection of hepatic metastases by use of jackknife free-response receiver-operating characteristic (JAFROC) analysis.	HCC, Metastasis, or hemangioma	74	Histological examination after surgical resection (10 lesions), US-guided biopsy (110 lesions), or contrast-enhanced CT and/or MRI for hemangioma	The integration of liver ultrasound cine clips with contrast enhancement and B-mode ultrasound improves detection sensitivity for hepatic metastases (41.6% to 72.2%), reduces false positives, and raises the figure of merit (0.44 to 0.76). The technique helps clinicians to diagnose tumors more accurately.
							33		
							30		
**Kume** **et al., 2010 [31]**	Japan	Prospective study	Not mentioned		To analyze the diagnostic accuracy of Sonazoid for detecting hepatic metastases from GIT cancers and to compare it with FDG PET-CT results.	Liver metastasis	109	In the cases with different results between CEUS and PET-CT, results of hepatic resection, dynamic CT, and/or magnetic resonance imaging, as well as followed-up clinical courses in some cases, were used for assistant diagnostic methods	Among 109 subjects, 34 had upper gastrointestinal tract cancer (esophageal 4, gastric 29, duodenal 1), and 76 had colorectal cancer (1 case was complicated with gastric cancer); the sensitivities and specificities of CEUS were 89.7% and 97.5%, respectively. In conclusion, CEUS with Sonazoid had similar efficacy in diagnosing hepatic metastasis as PET-CT.
**Sun et al.,** **2023 [19]**	China	Prospective study	56.0 (47.3–64.7)	21/8	To compare the diagnostic efficacy of SonoVue and S-CEUS in detecting colorectal liver metastasis (CRLM) after chemotherapy.	Colorectal liver metastasis	297	Histopathology for surgically removed lesions or intraoperative ultrasound with MRI for unresected liver lesions	In a study with 348 liver lesions, including 297 colorectal metastases, SonoVue showed significantly better diagnostic accuracy (64.7% vs. 54.0%) and sensitivity (63.3% vs. 50.5%) than Sonazoid. Both methods had similar specificity (72.5% vs. 74.5%). Notably, 40 metastases were mischaracterized by Sonazoid as benign lesions. The conclusion is that SonoVue performs better in diagnosing colorectal metastases after chemotherapy.
							297		
**Kobayashi et al., 2017 [20]**	Japan	Retrospective study	66.4 (58.0–74.8)	62/36	The study aimed to verify the effectiveness of S-CEUS in detecting, diagnosing, and providing additional benefits in the management of patients with liver tumors suspected to be metastatic.	Liver metastasis	121	Histopathological examination	S-CEUS had 95.0% sensitivity, 44.4% specificity, and 85.8% accuracy in the diagnosis of metastases. Accuracy was diminished by increased BMI and lesion depth, and management was impacted by S-CEUS in 8.2% of patients. The addition of S-CEUS improves diagnostic quality and decision-making for liver metastasis assessment.
**Mishima et al.,2016 [22]**	Japan		59 (48.0–68.0)	Females Only	To assess the effectiveness of Sonazoid-enhanced ultrasonography in detecting hepatic metastases in breast cancer patients. comparing the clinical efficacy and sensitivity of this technique with conventional contrast-unenhanced B-mode ultrasonography.	Liver metastasis	84	CT and MRI	In a study with 79 nodules, Sonazoid-enhanced ultrasonography (SEUS) showed high sensitivity (98.8%) and accuracy (98.7%) in detecting liver metastases in breast cancer patients, outperforming conventional B-mode ultrasound. SEUS had lower false positive and false negative rates and detected additional tumors. The study concluded that Sonazoid is safe, and that SEUS is more effective than B-mode ultrasound, particularly for small lesions under 14 mm in diameter.
**Correas et al.,** **2011 [29]**	Austria	Prospective study	62.4 (52.3–72.5)	1.4/1	To analyze perfluorobutane microbubble (NC100100) CEUS doses for optimal detection of liver metastases in patients with non-hepatic primary malignancies.	Liver metastasis	165	Contrast-enhanced CT and MRI	In a study with 92 patients and 165 liver metastases, contrast-enhanced ultrasound (CEUS) at a 0.12 μL/kg dose demonstrated significantly higher sensitivity (78%) and accuracy (70%) compared to other doses, highlighting the dose-dependent nature of CEUS for detecting liver metastases.
							32		
							40		
							36		
							57		
**Shiozawa et al., 2016 [35]**	Japan	Prospective study	66.0 (54.8–77.2)	1/2	To compare contrast-enhanced ultrasonography (CEUS) using Sonazoid with Gd-EOB-DTPA-enhanced MRI (EOB-MRI) in the diagnosis of liver metastases in patients with colorectal cancer.	Liver metastasis	109	Histopathology and imaging	In the study, both contrast-enhanced ultrasound (CEUS) and EOB-MRI detected liver metastases, with CEUS showing higher specificity (84.5% vs. 70.8%) and positive predictive value (97.1% vs. 93.7%) compared to EOB-MRI. Overall diagnostic accuracy was 90.2% for CEUS and 91% for EOB-MRI. The conclusion is that CEUS is more specific for diagnosing liver metastases than EOB-MRI.
**Ishikawa et al.,** **2019 [30]**	Japan	Prospective study	Not mentioned	26/15	To assess the diagnostic efficacy of contrast-enhanced endoscopic ultrasonography (CE-EUS) using Sonazoid for pancreatic cancer, with a focus on liver metastases, comparing the results with contrast-enhanced transabdominal ultrasonography (CEUS) following CE-EUS and Gd-EOB-DTPA-enhanced MRI (EOB-MRI).	Liver metastasis	30	Histopathological examination	In the study, patients undergoing contrast-enhanced endoscopic ultrasonography (CE-EUS) for pancreatic cancer were analyzed. EOB-MRI demonstrated 100% sensitivity, 88.2% specificity, and 93.7% positive predictive value (PPV) for liver metastases, while CEUS showed 83.3% sensitivity, 100% specificity, and 100% PPV. CEUS, especially valuable in differentiating liver metastasis from abscess, exhibited higher specificity and PPV than EOB-MRI.
**Oba et al.,** **2018 [23]**	Japan		59.0 (43.5–74.5)	71/29	To investigate the clinical implications of disappearing liver metastases from colorectal cancer after chemotherapy in the era of modern imaging studies.	Colorectal liver metastasis	136	Histological examination	In 59 patients with 275 colorectal liver metastases (DLMs), 26% of lesions were identified by EOB-MRI, 92% of which were viable at the time of resection. Together, EOB-MRI and CE-IOUS identified and resected 60% of DLMs, 77% of which contained viable disease. Non-resected lesions had a recurrence rate of 14% at 27 months. Sequential use of EOB-MRI and CE-IOUS effectively detect clinically significant DLMs.
**Takahashi et al., 2012 [38]**	Japan	Prospective study	63.0 (48.6–77.4)	67/35	To evaluate the effectiveness of contrast-enhanced intraoperative ultrasonography (CE-IOUS) using lipid-stabilized perfluorobutane microbubbles in detecting and enumerating colorectal liver metastases.	Colorectal liver metastasis	381	Histopathology for resected specimens and the results of follow-up studies using CT and/or MRI carried out within 3–6 months after the operation	In a study of 102 patients with 315 lesions, contrast-enhanced intraoperative ultrasound (CE-IOUS) identified more lesions than conventional examination. CE-IOUS showed 97.1% sensitivity, 59.1% specificity, and 93.2% accuracy, leading to a change in the surgical plan in 14.7% of cases. The conclusion is that CE-IOUS provides additional information beyond preoperative imaging and conventional intraoperative examinations.
**Tani et al.,** **2017 [37]**	Japan	Retrospective study	63.2 (49.0–77.4)	10:10	To assess the detectability of EOB-MRI and CE-IOUS for residual disease in disappearing colorectal liver metastases (DLMs) and to explore optimal management strategies for DLMs.	Colorectal liver metastasis	619	Histopathology and various imaging modalities	In the study, EOB-MRI outperformed CE-IOUS in detecting residual tumors for colorectal liver metastases (DLMs). EOB-MRI had higher accuracy in predicting residual disease (0.90 vs. 0.70). Of the undetected lesions by CE-IOUS that regrew after surgery, 81.8% were identified on EOB-MRI. The conclusion suggests the superiority of EOB-MRI for detecting residual tumors, emphasizing the need for maximum resection attempts for lesions visualized in EOB-MRI.

**Table 2:** The characteristics of studies are included only in the process of qualitative evidence synthesis.

Study ID	Country	Design	Age (Mean)	Sex (M/F)	Aim	Type of Lesions	Number of Lesions	Reference Test	Conclusion and Results
**Araki et al.,** **2019 [39]**	Japan	Cohort	55	2/1	In this article, the authors reported the significance of RVS-CEUS as a novel technique in hepatic resection for detecting small liver lesions.	Liver metastasis	10	Dynamic CT and EOB-MRI	The study found that intraoperative real-time virtual sonography with contrast-enhanced ultrasound (RVS-CEUS) had a 90% detection rate for small liver lesions (<10 mm) post-chemotherapy, outperforming CE-CT (50%) and EOB-MRI (100%). RVS-CEUS detected lesions as small as 3.0 mm with a maximum depth of 43.5 mm, making it a promising technique for hepatic resection.
**Hakamada et al., 2011 [42]**	Japan	Prospective study	Not mentioned		Evaluated Sonazoid-enhanced CE-IOUS for visualizing colorectal liver metastases (CRLM) vascularity and Kupffer cells, comparing its performance with dynamic CT, MR, and PET-CT. Aimed to enhance the precision of detecting smaller lesions in the context of evolving chemotherapy and targeted therapy for initially unresectable CRLM.	Colorectal liver metastasis	59	Intra-operative (patients undergoing hepatectomy)	CE-IOUS with Sonazoid detected tumors in the post-vascular phase at 92.5%, surpassing conventional US (75.4%), CT (85.5%), EOB-MRI (92.5%), DWI-MRI (85.7%), and PET-CT (51.8%)—additional detection by CE-IOUS altered procedures in 15.6% of patients, facilitating R0 resections. Conclusively, CE-IOUS with Sonazoid is a valuable tool for assessing smaller colorectal liver metastases undetected by conventional modalities, potentially enhancing patient outcomes.
**Uchiyama et al.,** **2010 [51]**	Japan	?	71.8	3/5	The study aimed to assess the efficacy of combining CE-IOUS and fluorescence navigation (photo dynamic eye with indocyanine green) for identifying colorectal metastatic lesions. This was compared with preoperative contrast-enhanced CT and gadoxetic acid-enhanced MRI.	Colorectal liver metastasis	52	Histopathology of biopsy or resected tissue	In the study, 56 lesions were identified, with 52 confirmed as metastases and 4 as benign tumors. Metastases detected by CE-IOUS and PDE [51] were higher than those by MDCT and EOB-MRI [46]. The combined use of CE-IOUS and PDE improved diagnostic sensitivity compared to MDCT and EOB-MRI (98.1% vs. 88.5%). The conclusion is that concomitant use of CE-IOUS with Sonazoid and the PDE system is a useful and safe method in addition to CT or MRI for identifying metastases.
**Minami et al., 2010 [52]**	Japan	Retrospective	65.8	51/15	Conventional contrast harmonic sonography has a technical problem of a short enhancement time during the targeting of hepatic malignancies for radiofrequency (RF) ablation. This study investigated the effectiveness of contrast harmonic sonographic guidance using perfluorocarbon microbubbles (Sonazoid) during RF ablation of hepatic malignancies.	HCC and secondary liver malignancies	108	CT	Findings: Based on sonography, the maximum diameters of all the tumors varied from 0.7 to 3.5 cm (mean ± SD, 1.7 ± 0.9). In 62 (94%) of the 66 patients, complete tumor necrosis was attained with a single RF ablation treatment. For the remaining four patients (6%), two sessions were needed. There were 1.1 0.3 therapy sessions on average. Of the 108 malignant hepatic tumors, 105 (97%) had a defect with a margin during the post-vascular period. During a follow-up period of 1–12 months (mean, 4.3 months), the clinical courses were excellent, with no evidence of local tumor progression.
**Uetake et al., 2012 [50]**	Japan	Cohort	_	_	Chemotherapy has changed the strategy for hepatic metastasis. Unresectable hepatic metastases can become resectable after chemotherapy. Contrast-enhanced intraoperative ultrasonography (CE-IOUS) helps to detect undetectable hepatic lesions. Even shrunken tumors have viable cancer cells, and CE-IOUS helps to detect minute foci.	Colorectal cancer	57	Preoperative CT and pathological examination	Before chemotherapy, 57 metastatic lesions were found. CE-IOUS used perflubutane to demonstrate thirty lesions, which were subsequently excised. Twelve of the samples did not show tumor cells in the pathological investigation. There were thirty resected lesions in total. All patients had pathological liver damage of grade 1 or less, and none of them experienced any major postoperative complications.
**Ueda et al.,** **2016 [49]**	Japan	Retrospective	73.8	3/2	This study investigated whether changes in the time–intensity curve (TIC) of CEUS are useful indicators of the therapeutic effect of chemotherapy.	Rectal, gastric, and esophageal	_	CT	Out of the five TIC parameters that were investigated, the area under the curve and the ROC of the wash-in slope indicated that the therapeutic impact of chemotherapy was superior to the other three characteristics. (ii) Two of the five patients had their TIC parameters evaluated following a single chemotherapy cycle, and modifications in the area under the curve and the slope of washing were in good agreement with computed tomography results that indicated the therapeutic impact following the fourth chemotherapy cycle.
**Tochio et al., 2015 [48]**	Japan	Cohort	HER-positive: 67, HER-negative: 62	HER-positive: 6/2 HER-negative: 4/1	A hyper-enhanced rim (termed “HER”) in the post-vascular phase is detected in some cases of liver metastasis by Sonazoid-enhanced ultrasonography (US). Here, the association of the HER with histological features was investigated to clarify the cause of this characteristic imaging pattern.	Liver metastasis	13	Histopathology	The distribution density of CD68-positive cells in the HER-positive group (*n* = 8) was 2.9 ± 0.9, substantially greater than that in the HER-negative group (1.0 ± 0.3) (*p* < 0.05). In the vicinity of the tumor, inflammatory cell infiltrates, including CD8-positive lymphocytes, were found in all HER-positive instances, whereas fibrosis was noted in all HER-negative cases. In the HER-negative group, the tumor’s necrotic region was noticeably bigger.
**Ramnarine et al., 2000 [47]**	UK	Cohort	Age range of, 38–77 years	_	To assess the vascularization of focal hepatic tumors using NC100100 dynamic power Doppler imaging and to quantify the power Doppler signal intensity (PDSI) to provide contrast agent wash-in (PDSI–time) curves of focal liver lesions.	Liver metastasis	22	Histopathology	The PDSI–time curves within hemangiomas were flat, while the PDSI increased quickly in liver metastases. Of the participants with liver metastases, eleven had a significant rise in PDSI within the tumor, whereas just one had a hemangioma. Four patients had hemangiomas visible surrounding an improved rim. The dose of the contrast agent and the peak PDSI inside metastases did not appear to be correlated.
**Patel et al.,** **2010 [46]**	Japan	Cohort	25 to 85	11/24	To compare Sonazoid-enhanced ultrasound (SEUS) and contrast-enhanced computed tomography (CECT) enhancement and washout patterns in hepatic lesions.	HCCs_(ICCs)_metastatic lesions_FNHs_UBLs_AMLs	61	Histopathology	On both SEUS and CECT, all 61 lesions (100%) exhibited vascular enhancement. For the 61 lesions, there was a 93.4% “washout/no washout” agreement between SEUS and CECT (j coefficient: 0.816). There were 42 malignant lesions in total. Washout was seen in 38 lesions (90.5%) on both SEUS and CECT. Washout was seen on SEUS but not on CECT in the remaining four malignant lesions, three of which had fibrosis (two intrahepatic cholangiocarcinoma and one scirrhous hepatocellular carcinoma). The agreement between SEUS and CECT for the 19 benign lesions was 100% (j coefficient: 1), with 7 lesions exhibiting washout using both techniques and 12 lesions exhibiting no washout using either technique.
**Nanashima et al., 2010 [45]**	Japan	Case-control	68.1	34/16	To elucidate different facets of IOUS. To achieve this, we evaluated the contrast-media IOUS employing Sonazoid in 50 hepatectomy patients who had liver tumors, as well as the viability and constraints of this technique. The outcomes were compared historically with patients who had a hepatectomy between 2004 and 2006 using traditional IOUS.	HCC, ICC, or colorectal liver metastasis			The cancers included gastrointestinal stromal tumor in 1 case, benign hematoma in 1 case, colorectal liver metastases in 14 cases, intrahepatic cholangiocarcinoma in 3 cases, and hepatocellular carcinoma (HCC) in 25 individuals. In the majority of instances, liver tumors were easily identified as perfusion defects. On the Sonazoid IOUS, small lesions (<1 cm), extra-capsular tumor development, and portal vein tumor thrombus were also plainly seen. In five cases, small occult tumors were discovered. Early vascular findings allowed for a differential diagnosis with suspected non-tumorous lesions and benign masses. The proportion of patients with positive surgical margin (0%) tended to be lower than that of the control group (P¼0.073) when compared to hepatectomy for HCC under traditional IOUS.
**Nakano et al.,** **2008 [44]**	Japan	Brief clinical report			To detect occult metastases during hepatectomy in patients with colorectal cancer liver metastases (CRCLM). Sonazoid, a novel microbubble agent that produces a parenchyma-specific contrast image based on its accumulation in the Kupffer cells, was used in contrast-enhanced intraoperative ultrasound sonography (CE-IOUS).	Colorectal liver metastasis		CT and MRI	Sonazoid was injected into CE-IOUS, which produced early vascular and sinusoidal phase images for ten minutes and late Kupffer phase images for up to thirty minutes. The eight patients’ metastatic lesions were not found to be new by IOUS. However, in two of the eight patients, a new metastatic lesion was discovered during the late Kupffer-phase Sonazoid imaging. These recently discovered tumors were removed during a second hepatectomy, and histological analysis revealed that they were metastases.
**Edey et al.,** **2008 [40]**			67.3	10/16	To determine whether phase-inversion imaging and delayed-phase liver imaging with a destructive imaging mode can yield information that is comparable to phase-inversion imaging in terms of liver metastasis detection and conspicuity.	Liver metastasis		Histopathology after biopsy of a focal lesion or tissue resection	Sixteen patients were included (ten men and six women), with a mean age of 67.3 years (range: 48–83 years). Group A n ¼ 1, Group B n ¼ 8, Group CI n ¼ 1, Group CII n ¼ 4, and Group CIII n ¼ 2 were the divisions based on CECT imaging. The committee agreed that baseline ultrasonography was accurate at 75% compared to CECT in 12 out of 16 patients, while contrast-enhanced ultrasound (CEUS) was accurate at 93.8% (15/16). When comparing low (*p* = 0.0029) and high MI phase-inversion (*p* = 0.0004) and destructive (*p* = 0.0015) CEUS imaging to baseline ultrasonography, there was a significant improvement in lesion conspicuity. At greater Sonazoid doses, artifacts were observed; no adverse effects were reported.
**Hiroyoshi** **et al., 2021 [41]**	Japan	Prospective study	70	11/6	This prospective study aimed to assess CE-IOUS’s effectiveness in predicting Glisson’s capsule invasion during hepatectomy for CLM based on the three parameters listed above.	Colorectal liver metastasis	24	CT and EOB-MRI	Pathological tests revealed Glisson invasion in 24 (13%) of the 187 CLMs removed. In three tumors (1.6%), peripheral dilatation was seen by IOUS; in four tumors (2.1%), border irregularity and/or caliber change were found in twenty-four tumors (12.8%). Preoperative EOB-MRI revealed Glisson invasion in only four tumors (sensitivity/specificity, 17%/100%). However, IOUS had a 79% and 96% sensitivity and specificity for diagnosing Glisson invasion with any of the three findings mentioned above. By using receiver operating characteristic analysis, the cutoff value of caliber change for the diagnosis of Glisson invasion was established at 140%. There was no significant difference in the R0 resection rates between patients who had Glisson invasion (85%) and those who did not (82%).
**Itabashi et al., 2013 [43]**	Japan	Prospective study	64.5	41/30	The study aimed to evaluate the clinical value of Sonazoid-enhanced intraoperative laparoscopic ultrasonography (S-IOLUS) in patients undergoing laparoscopy-assisted colectomy (LAC) for colorectal cancer. This approach addresses the limitations of the conventional palpation and observation of the liver during LAC.	Colorectal cancer		Preoperative imaging, CE-CT, and/or MRI	S-IOLUS (*n* = 71) found minor hypoechoic lesions in the Kupffer-phase image of two individuals, despite IOLUS not finding any lesions (2.8%). Within six months following LAC, none of the 71 patients who had S-IOLUS revealed liver metastases. Two patients (2.6%) in the traditional IOLUS group (*n* = 77) had metastatic lesions found in them. Six months following LAC, the two patients’ fresh liver metastases were found. The new liver metastases in these two patients were detected within 6 months after LAC.

## Data Availability

Data that support the findings presented in this manuscript will be made available upon reasonable request.

## Supplementary Materials

The following supporting information can be downloaded at: https://www.mdpi.com/article/10.3390/medsci13020042/s1, Figure S1: CEUS SROC curve in detecting metastatic lesions; Figure S2: CIOS SROC curve in detecting metastatic lesions; Figure S3: Subgroup analysis for CEUS sensitivity and specificity in detecting liver metastatic lesions; Figure S4: Subgroup analysis for CEUS 3D sensitivity and specificity in detecting liver metastatic lesions; Table S1: The detailed search strategy; Table S2: The reasons for the exclusion of studies; Table S3: The quality assessment of the included articles [18,19,20,21,22,29,30,31,32,33,34,35,36,39,40,41,42,43,44,45,46,47,48,49,50,51,52,53,58,59,60,61,62,63,64,65,66,67,68,69,70,71,72,73,74].
