# Supplementary material for: Diagnostic Accuracy of Sonazoid-Enhanced Ultrasonography for Detection of Liver Metastasis
Source: Med Sci (Basel). 2025 Apr 9;13(2):42. doi: 10.3390/medsci13020042 (PMC12015772; doi:10.3390/medsci13020042)
Supplement: Supplementary file 1 [file medsci-13-00042-s001.zip › medsci-3496048-supplementary.pdf]

Supplementary Table S1. The detailed search strategy.

<table>
<tr><th>Databases</th><th>Restrictions</th><th>Term</th><th>Items found</th></tr>
<tr><td>PubMed</td><td>Restricted to title and abstract</td><td rowspan="5">((((Ultrasound OR Echotomography OR ultrasonic OR Ultrasonograph* OR Sonograph* OR Sonogram OR Echography OR "U/S" OR US OR echoscopy OR Ultrasoun* OR "Ultrasonic Tomography")) AND ((Sonazoid OR NC100100 OR DD-723 OR decafluorobutane OR "nc 100100" OR "perfluoro n butane" OR perfluorobutane OR "Perfluoro-n-butane"))) AND ((Hepatocellular OR Hepatic OR Liver))) AND ((metastasis OR Metastases OR Metastase OR metastatic OR "secondary tumor" OR spread OR migration)).</td><td>51</td></tr>
<tr><td>Embase</td><td></td><td>208</td></tr>
<tr><td>Web of Science</td><td>Restricted to topic</td><td>66</td></tr>
<tr><td>Scopus</td><td rowspan="2">Restricted to title, abstract and keyword</td><td>143</td></tr>
<tr><td>Cochrane</td><td>5</td></tr>
<tr><td>Total</td><td></td><td></td><td>473</td></tr>
</table>

Supplementary Table S2. The reasons for exclusion of studies

Studies excluded	Reasons
Sugimoto,2017[58]	No Liver metastasis
Mansour,2021[59]	No Sonazoid
Minami,2014[60]	literature review
Muaddi,2021 [61]	Systematic review
Okada,2002[62]	Not HCC or Liver Met. Patients
Ottomo2011 [63]	Case report
sugimoto2009 [64]	Computer-aided with CEUS
sugimoto2016 [65]	No Liver metastasis (pilot study)
Tranquart2022 [66]	(letter to editor)
watanabe2013[67]	Case report
Wilson,2001[68]	literature review
Wu2022 [69]	(protocol)
Xu2023[70]	Ultrasound-guided laparoscopic radiofrequency
Kita 2012 [71]	No Liver metastasis
Nuta 2016 [72]	No Liver metastasis
Hiraoka 2009 [73]	No Liver metastasis
Hatanaka 2010 [74]	No Liver metastasis

Supplementary Table S3. The quality assessment of included articles.

Study ID	C1	C2	C3	C4	C5	C6	C7	C8	Final score	comment
Araki 2019 [39]	+	+	+	-	+	+	-	+	6	Good
Edey 2008 [40]	+	+	+	-	+	+	+	+	7	Good
Goto 2009[53]	+	+	+	-	-	+	-	+	5	Fair
Hiroyoshi 2020 [41]	+	+	+	-	+	+	-	+	6	Good
Hakamada 2011[42]	+	+	+	-	-	+	-	+	5	Fair
Itabashi 2013 [43]	+	+	+	-	+	+	-	+	6	Good
Nakano 2008 [44]	+	-	+	-	+	+	-	+	5	Fair
Nanashima 2010 [45]	+	+	+	-	+	+	-	+	6	Good
Patel 2009 [47]	+	+	+	-	+	+	-	+	6	Good
Ramnarine 2000 [47]	+	+	+	-	+	+	-	+	6	Good
Tochio 2015 [48]	+	-	+	-	+	+	-	+	5	Fair
Ueda 2016 [49]	+	+	+	-	+	+	-	+	6	Good
Uetake 2012 [50]	+	+	+	-	+	+	-	+	6	Good
Uchiyama 2010 [51]	+	+	+	-	+	+	-	+	6	Good
Minami 2010 [52]	+	+	+	-	+	+	-	+	6	Good

Supplementary Table S2. Shows quality assessment of all included cohort and cross-sectional studies using the National Institute of Health (NIH) quality assessment.

C1: Was the research question or objective in this paper clearly stated?
C2: Was the study population clearly specified and defined?
C3: Were all the subjects selected or recruited from the same or similar populations (including the same time period)? Were inclusion and exclusion criteria for being in the study prespecified and applied uniformly to all participants?
C4: Was a sample size justification, power description, or variance and effect estimates provided?
C5: Were the exposure measures (independent variables) clearly defined, valid, reliable, and implemented consistently across all study participants?
C6: Were the outcome measures (dependent variables) clearly defined, valid, reliable, and implemented consistently across all study participants?
C7: Were the outcome assessors blinded to the exposure status of participants?

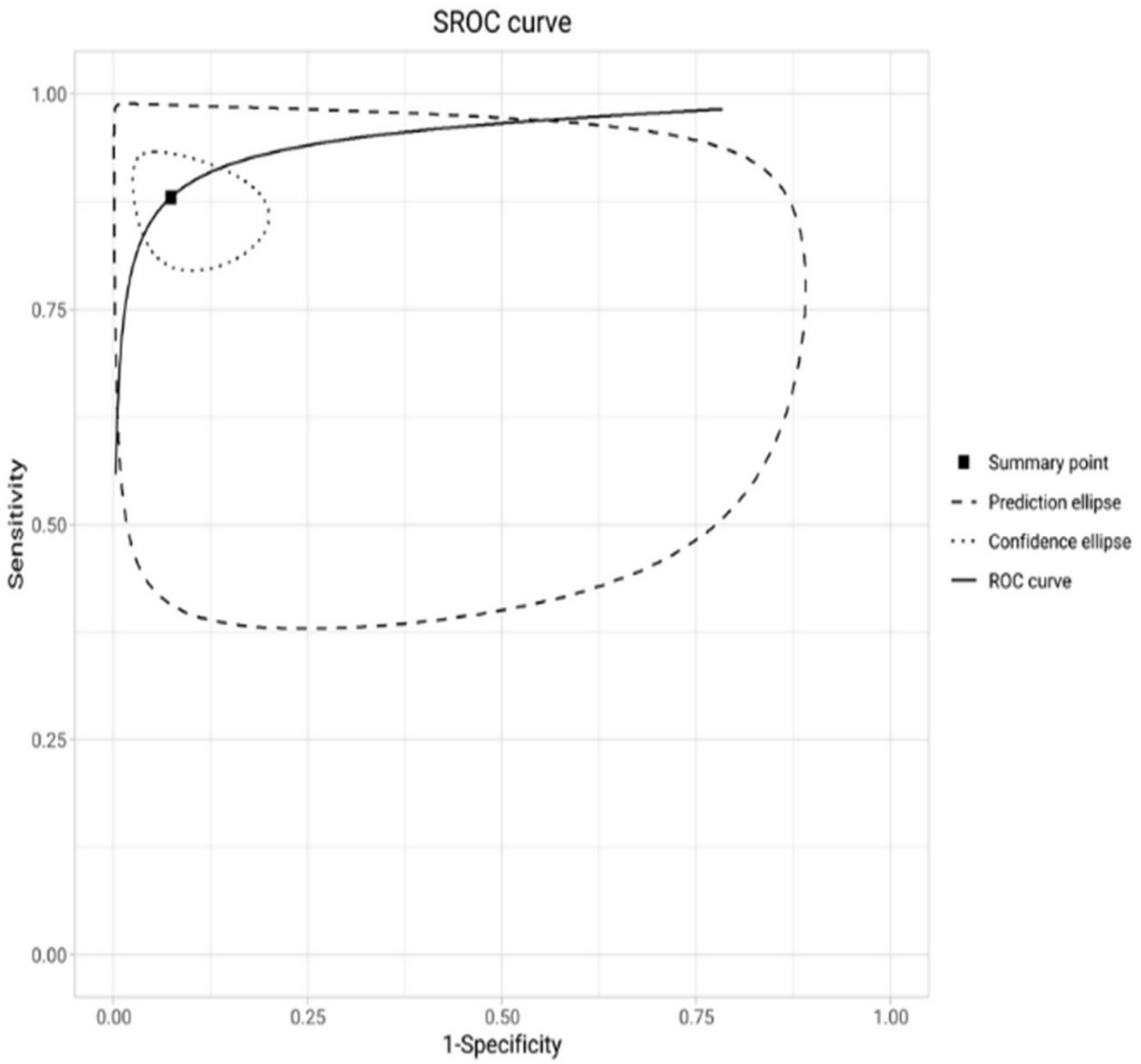

Supplementary Figure S1: CEUS SROC curve in detecting metastatic lesions.

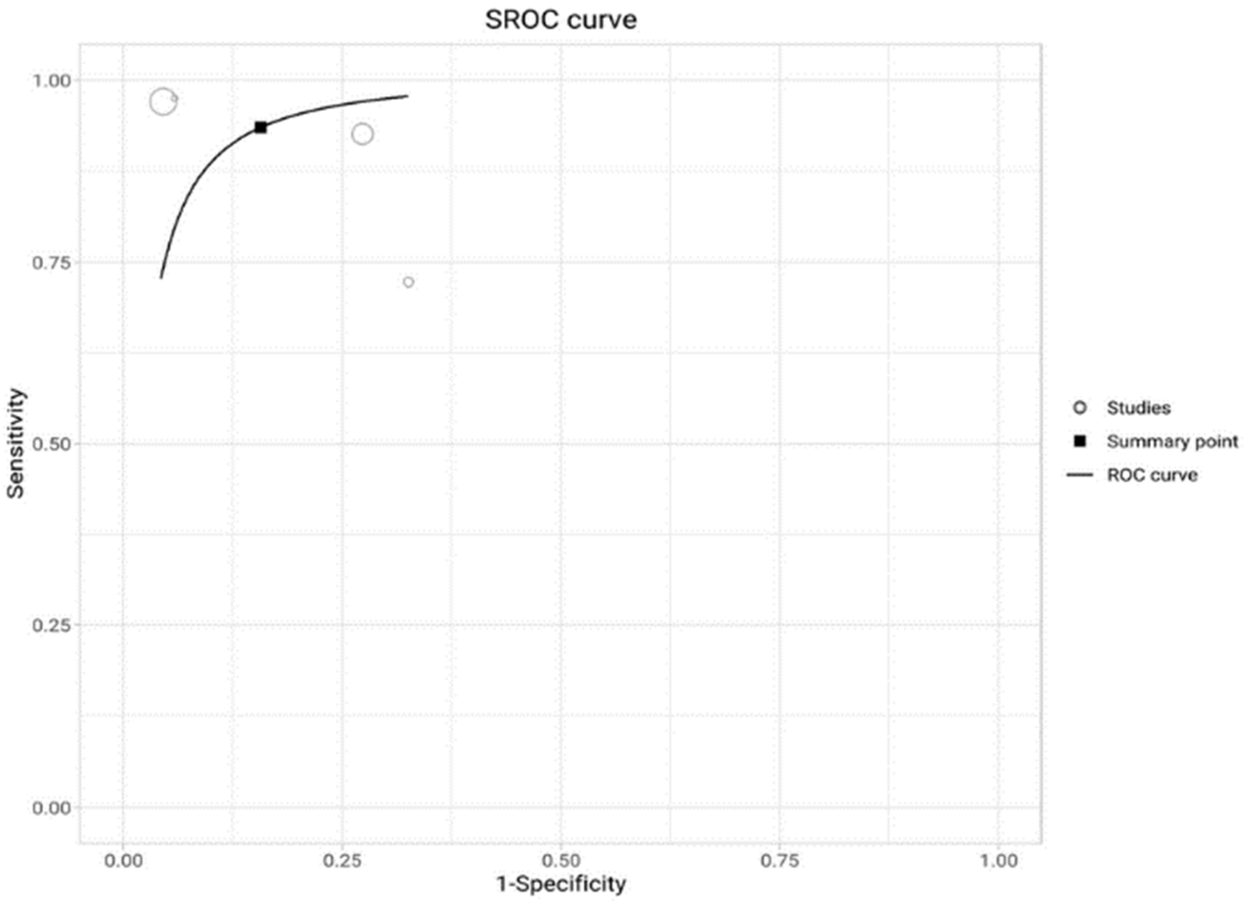

Supplementary Figure S2: CIOS SROC curve in detecting metastatic lesions.

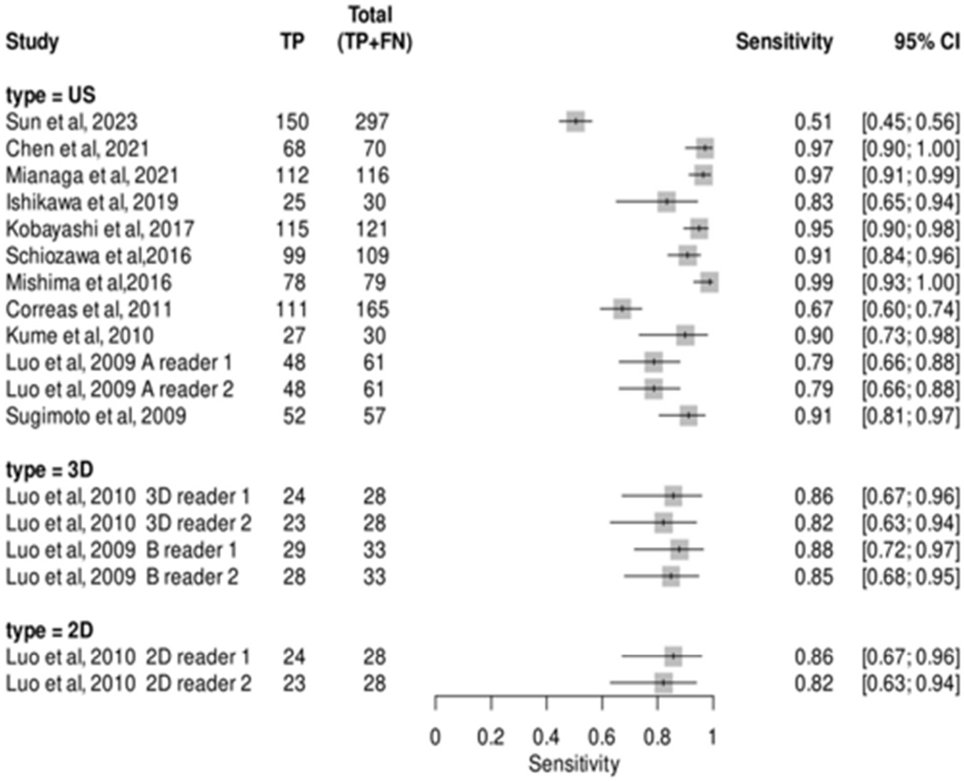

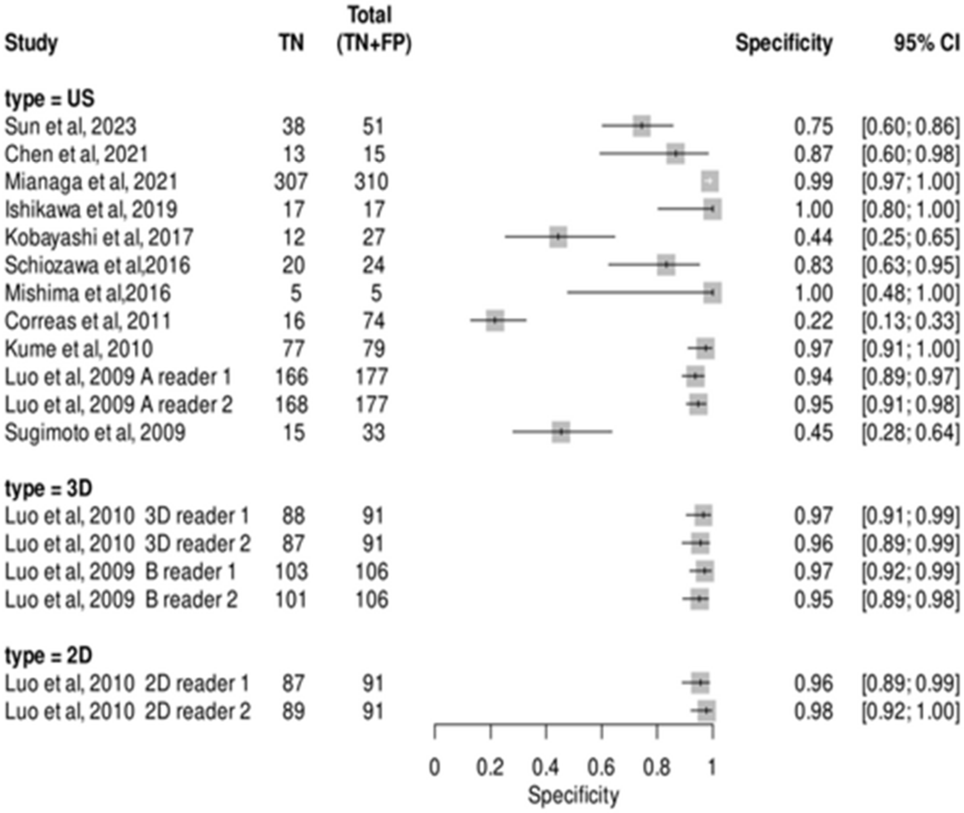

Supplementary Figure S3: Subgroup analysis for CEUS sensitivity and specificity in detecting liver metastatic lesions. [18–22,29–36]

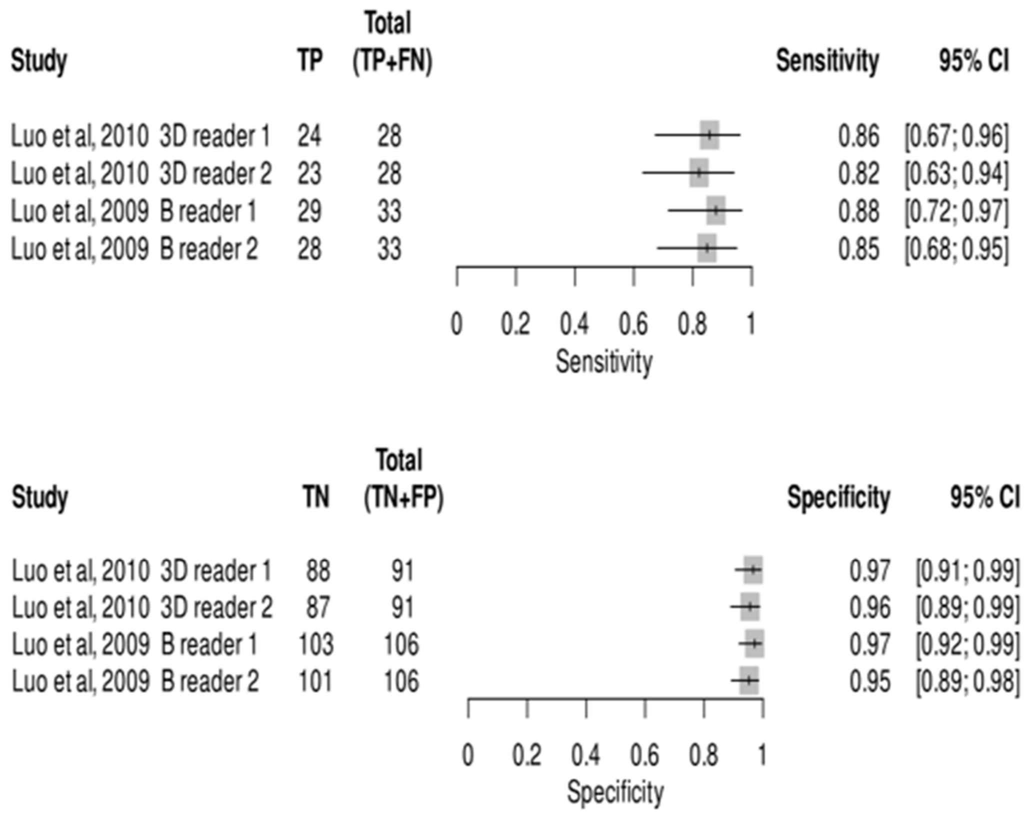

Supplementary Figure S4: Subgroup analysis for CEUS 3D sensitivity and specificity in detecting liver metastatic lesions. [21, 32]
